# Cost of management of severe pneumonia in young children: systematic analysis

**DOI:** 10.7189/jogh.06.010408

**Published:** 2016-06

**Authors:** Shanshan Zhang, Peter M. Sammon, Isobel King, Ana Lucia Andrade, Cristiana M. Toscano, Sheila N Araujo, Anushua Sinha, Shabir A. Madhi, Gulam Khandaker, Jiehui Kevin Yin, Robert Booy, Tanvir M Huda, Qazi S Rahman, Shams El Arifeen, Angela Gentile, Norberto Giglio, Mejbah U. Bhuiyan, Katharine Sturm–Ramirez, Bradford D. Gessner, Mardiati Nadjib, Phyllis J. Carosone–Link, Eric AF Simões, Jason A Child, Imran Ahmed, Zulfiqar A Bhutta, Sajid B Soofi, Rumana J Khan, Harry Campbell, Harish Nair

**Affiliations:** 1 Usher Institute of Population Health Sciences and Informatics, University of Edinburgh, Edinburgh, UK; 2 Department of Preventive Dentistry, Peking University, School and Hospital of Stomatology, Beijing, PR China; 3 NHS Grampian, UK; 4 Department of Community Health, Federal University of Goias, Brazil; 5 State University of Maranhăo, Brazil; 6 New Jersey Medical School, Rutgers, The State University of New Jersey, Newark, New Jersey USA; 7 Medical Research Council, Respiratory and Meningeal Pathogens Research Unit, Faculty of Health Sciences, University of the Witwatersrand, Johannesburg, South Africa; 8 Department of Science and Technology/National Research Foundation, Vaccine Preventable Diseases, University of the Witwatersrand, Johannesburg, South Africa; 9 National Centre for Immunisation Research and Surveillance, The Children's Hospital at Westmead, NSW, Australia; 10 Sydney School of Public Health, Faculty of Medicine, The University of Sydney, NSW, Australia; 11 Centre for Child and Adolescent Health, icddr,b, Dhaka, Bangladesh; 12 School of Public Health, Sydney Medical School, University of Sydney, Sydney, Australia; 13 Epidemiology Department, Ricardo Gutierrez Children Hospital, University of Buenos Aires, Argentina; 14 Centre for Communicable Diseases, icddr,b, Dhaka, Bangladesh; 15 Influenza Division, National Center for Immunization and Respiratory Diseases, Centers for Disease Control and Prevention, Atlanta, GA, USA; 16 Agence de Médicine Préventive, Paris, France; 17 Faculty of Public Health, University of Indonesia, Jakarta, Indonesia; 18 Department of Pediatrics, Section of Infectious Diseases, University of Colorado Denver School of Medicine, Denver, CO, USA; 19 Center for Global Health and Department of Epidemiology, Colorado School of Public Health, Aurora, CO, USA; 20 Pharmacy Department, Children's Hospital Colorado, Aurora, CO, USA; 21 Center of Excellence in Women and Child Health, the Aga Khan University, Karachi, Pakistan; 22 Department of Paediatrics & Child Health, The Aga Khan University, Karachi, Pakistan; 23 James P Grant School of Public Health, BRAC University, Dhaka, Bangladesh; 24 Public Health Foundation of India, New Delhi, India

## Abstract

**Background:**

Childhood pneumonia is a major cause of childhood illness and the second leading cause of child death globally. Understanding the costs associated with the management of childhood pneumonia is essential for resource allocation and priority setting for child health.

**Methods:**

We conducted a systematic review to identify studies reporting data on the cost of management of pneumonia in children younger than 5 years old. We collected unpublished cost data on non–severe, severe and very severe pneumonia through collaboration with an international working group. We extracted data on cost per episode, duration of hospital stay and unit cost of interventions for the management of pneumonia. The mean (95% confidence interval, CI) and median (interquartile range, IQR) treatment costs were estimated and reported where appropriate.

**Results:**

We identified 24 published studies eligible for inclusion and supplemented these with data from 10 unpublished studies. The 34 studies included in the cost analysis contained data on more than 95 000 children with pneumonia from both low– and–middle income countries (LMIC) and high–income countries (HIC) covering all 6 WHO regions. The total cost (per episode) for management of severe pneumonia was US$ 4.3 (95% CI 1.5–8.7), US$ 51.7 (95% CI 17.4–91.0) and US$ 242.7 (95% CI 153.6–341.4)–559.4 (95% CI 268.9–886.3) in community, out–patient facilities and different levels of hospital in–patient settings in LMIC. Direct medical cost for severe pneumonia in hospital inpatient settings was estimated to be 26.6%–115.8% of patients’ monthly household income in LMIC. The mean direct non–medical cost and indirect cost for severe pneumonia management accounted for 0.5–31% of weekly household income. The mean length of stay (LOS) in hospital for children with severe pneumonia was 5.8 (IQR 5.3–6.4) and 7.7 (IQR 5.5–9.9) days in LMIC and HIC respectively for these children.

**Conclusion:**

This is the most comprehensive review to date of cost data from studies on the management of childhood pneumonia and these data should be helpful for health services planning and priority setting by national programmes and international agencies.

Pneumonia is one of the leading causes of morbidity and mortality in children under–five globally, and accounted for about 935 000 (15%) deaths in 2013 and 120 million new episodes of illness in this age group in 2010 [1,2]. Appropriate management of childhood pneumonia can reduce pneumonia–specific mortality by 32–72% [3–5] and thus accelerate the progress toward achievement of the Millennium Development Goal 4 (MDG4). Childhood pneumonia places a large economic burden on families and the health care system, especially in resource–constrained low– and middle–income countries (LMIC). Severe ALRI is a substantial burden on health services worldwide and a major cause of hospital referral and admission in young children [6]. Although several studies in high–income as well as low– and middle–income countries have reported the costs associated with an episode of pneumonia (at the individual patient level), there are no published systematic reviews summarizing the evidence from different health systems and settings globally. Bahia et al reviewed pneumococcal disease costs and productivity loss in Latin America and the Caribbean showed variation in unit costs of pneumococcal pneumonia at outpatient and inpatient levels [7]. We aimed to conduct a systematic review of published data on the costs associated with management of pneumonia episodes in children younger than 5 years and to identify unpublished data sets from pneumonia research groups globally. Cost estimates based on these data should be useful to develop models for estimating cost of management of pneumonia in community as well as hospital–based settings.

## METHODS

### Review of published studies

We aimed to identify all published studies reporting empirical cost data on the treatment of episodes of pneumonia in children aged below 5 years during a 15–year period (1998–2013). We included studies in children younger than 5 years with pneumonia managed as in–patients or out–patients (using standard treatment per local standard) in secondary and tertiary hospitals, first level facility or in community settings. Data on the cost of a single episode of severe pneumonia from the societal and health care perspectives were collected as the primary study outcome. We developed a review protocol at the beginning of this study and followed the same throughout the process.

We undertook a systematic literature review with three reviewers (PS, IK, SZ), and hand searched reference list of all included articles. We searched four databases (with online search tools) to offer maximum coverage of the relevant literature: Medline, EMBASE, The Centre for Review and Dissemination Library (incorporating the DARE, NHS EED, and NHS HTA databases); and The Cochrane Library (via the Wiley Online Library) for the period 1 January 1998 to October 31 2013. (for search strategy, see Appendix S1 in **Online Supplementary Document(Online Supplementary Document)**).

Three review authors (SZ, PS, IK) independently selected potentially relevant studies based on their title and abstract. Any disagreements in study selection or data extraction were resolved after discussion with SZ and HC. The eligible studies were retrieved electronically for full–text review. We included studies that investigated all–cause pneumonia in a non–selective population sample, reported empirical cost data for pneumonia treatment (using any intervention including, but not limited to, antibiotics), and included only children younger than 5 years or reported data separately for this age group. We excluded review articles, vaccine cost–effectiveness trials, and studies considering specially selected cohorts with severe co–morbidity (Appendix S2 in **Online Supplementary Document(Online Supplementary Document)**). We developed and piloted a comprehensive data extraction template. We collected data on cost per episode, cost and unit cost of medication and services, duration of hospital stay and direct medical and non–medical costs. Direct medical cost included costs related to medication, diagnostic tests, medical staff time and hospital stay. Direct non–medical costs included those relating to food, transportation and accommodation charges. Any additional data on indirect costs such as care–givers’ time and earning loss were also recorded, where available. Additionally, we extracted data on study characteristics including country, treatment setting, study type and sample size. We recorded the cost study perspective only if explicitly stated in the text of the article to avoid subjective influence. For those papers that did not explicitly state the perspective used, we noted “–“ for “unstated”.

We classified countries into high income and low–and–middle income categories based on the classification adopted by the World Bank and according to 2012 Gross National Income (GNI) per capita, calculated using the World Bank Atlas method. The groups are low–income per capita US$ 1035 or less; lower middle–income US$ 1036–US$ 4085; upper middle income US$ 4086–US$ 12 615; and high income US $12 616 or more [8].

### Quality assessment

We assessed the quality of the included studies using a 13 point scale based on a modified Drummond checklist [9] for economic evaluation focusing on the methodological robustness and detail of reporting (Appendix S3 in **Online Supplementary Document(Online Supplementary Document)**). Studies were considered high quality if more than 10 points were addressed, medium quality studies covered 7–9 points and low quality studies addressed less than 6 points. Studies with all quality levels were included in the final analysis.

### Unpublished data collection

We collected unpublished data from 10 collaborating sites that were part of a Severe ALRI Working Group (SAWG) [6]. The study population included children under 5 years of age with a clinical diagnosis of pneumonia. We defined pneumonia using the World Health Organization’s (WHO) Integrated Mangement of Childhood Illness (ICMI) definition by three different severity categories: non–severe, severe and very severe pneumonia based on WHO pocket book for hospital care for children 2005 [10]. We included all interventions for pneumonia management as detailed in the WHO pocket–book (for community/and facility–based management) where data were available.

We designed a costing spreadsheet with detailed descriptions of case definitions and methods and used this for data collection. Actual cost of medications, supplies, personnel and average laboratory costs were collected. Methods used to gather primary cost data in these studies were recorded in the spreadsheet. Resource utilization data from patient records were also documented, where available, including length of stay in hospital, the quantity of drugs and supplies utilized by each patient, and the use of diagnostic tests and procedures. We also attempted to collect data on out–of–pocket spending (by patients) on transport and food where possible. Indirect cost of caregivers’ time and daily pay rate were also recorded. Primary data collection was conducted using the provided standardized templates and guidelines at individual study site. (Appendix S4 in **Online Supplementary Document(Online Supplementary Document)**).

**Figure 1:**
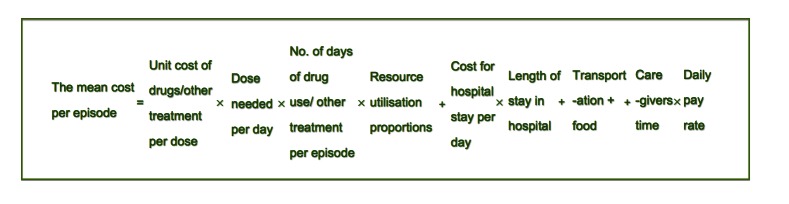
Formula for the mean total cost of treatment per episode.

We used a bottom–up approach to calculate cost per episode for each level of the intervention (community, first level health facility and hospital). Costs were calculated and presented separately based on severity and service delivery channels: very severe pneumonia at hospital level (defined as pneumonia with central cyanosis, inability to breastfeed or drink, or vomiting everything, convulsions, lethargy or unconsciousness and severe respiratory distress diagnosed by doctor or physicians using WHO IMCI (2005) case definition or pneumonia cases requires critical care); severe pneumonia at hospital level (defined as pneumonia with chest indrawing using WHO IMCI definition or pneumonia need for hospital admission based on physician’s assessment); severe pneumonia at community level (based on assessment by a trained health worker at home/first level facility using WHO IMCI (2005) case definition); and non–severe pneumonia at outpatient level (defined as fast breathing for age in children aged 2 to 59 months). The costing model included direct medical cost, direct non–medical cost and indirect costs. We calculated the cost per episode based on the estimates of the unit cost per contact (eg, unit cost of an antibiotic per day) at each management level multiplied by the resource utilization proportions (eg, 80% of children took amoxicillin for 5 days), plus indirect costs. For the mean total cost of treatment per episode we summed the cost of drugs, diagnostic investigations and hospital stay, as well as transportation and opportunity cost for caregivers’ time. The formula is given in **Figure 1**.

We reported all cost data in 2013 US$ equivalent prices. We first converted all costs to US$ and then adjusted for inflation to 2013 values. Conversions were made using the Penn World Tables 8.0 (http://www.ggdc.net/pwt) and an online inflation–calculating tool (http://usinflation.org/cpi-inflation-calculator) on 20^th^ October 2013.

### Statistical analysis

We have stratified the cost results by country income category: high–income countries (HIC) and low– and middle–income countries (LMIC). As an important input in the costing analysis for in–patient management, length of stay (LOS) in hospital was extracted for severe hospitalized cases. Cost per episode, cost by component (direct medical, direct non–medical and indirect costs), and percentage of total cost per episode in each component were summarized. Cost per episode was synthesized by severity of diseases in each strata. The mean with 95% CI of the treatment costs and the median with interquartile range (IQR) of LOS were estimated and reported where appropriate. The 95% CIs were calculated based on 5000 bootstrap samples. Mean and median values were compared using appropriate statistical tests.

Direct medical cost in studies reported from household perspective were compared with monthly household income in respective countries to evaluate the burden on families. Monthly household incomes were derived from Gallup World Poll using annual median household income divided by 12 months [11]. These income results were based on Gallup data gathered between 2006 and 2012 in 131 populations. In two countries which annual household income data was missing, we used GNI per capita from World Bank database times the mean number of people per household instead. The percentages of direct non–medical costs and indirect cost per episode of weekly household income were also assessed to show the economic impact of pneumonia management for families when direct medical cost was not considered.

We conducted all data analyses using SPSS v.19 (IBM, New York City, NY, USA) noting that included studies showed marked heterogeneity of population, methodology, treatment procedure reporting categories and perspectives.

## RESULTS

### Search results

**Figure 2:**
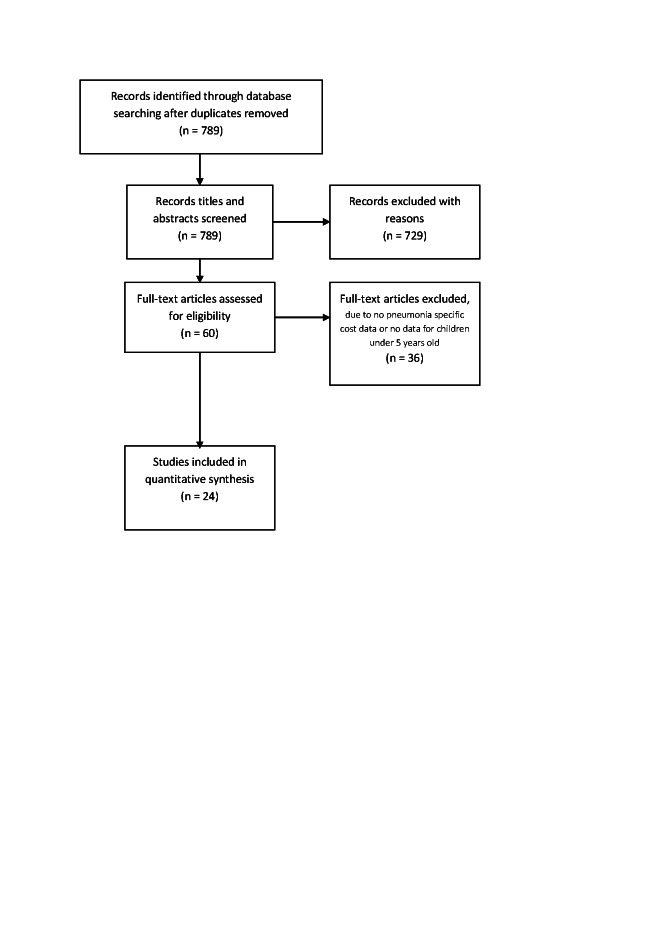
PRISMA flow diagram for severe pneumonia cost systematic review.

We identified 789 studies through database searching, of which 60 articles were eligible for full text review on the basis of title and abstract assessment (**Figure 2**). Subsequently, only 24 papers were identified to be eligible for data extraction and analysis. The key reasons for exclusions included: no data for children below 5 years or no cost data on pneumonia management were reported. For unpublished studies, we contacted 16 sites, 10 of which had data that met our eligibility criteria and contributed to the analysis. The unpublished cost data were for the period January 2001 to August 2012. Six of these sites provided cost data using a template and guidelines designed for this project while the remainder provided unpublished data in their own formats.

### Characteristics of published and unpublished data

We identified 24 studies from the literature review and collected additional 10 data sets of unpublished studies totalling 34 studies from 21 countries across the 6 WHO regions (**Table 1**). Over 60% of the studies (21 out of 34) were conducted in the South East Asia and Africa Regions. The included studies reported data from a variety of treatment settings: community, out–patient and in–patient care settings in primary, secondary and tertiary hospitals, and at city, district, provincial and national levels. Twenty–seven of the 34 studies were stand–alone primary cost analysis and/or cost–of–illness studies. The remaining 7 studies were designed to collect cost data alongside clinical trials or epidemiological studies.

The included studies reported cost data on a total of 97 062 children treated at facility or community levels, with a median sample size of 378 (IQR 117–741) across all studies. The age of the participants was reported in 12 studies and the median age was 12.3 months (IQR 8.20–33.20). The perspective of costing was explicitly stated in 30 of 34 studies. Of these, the most common perspectives were societal (16 out of 33, 1 study did not specify perspective), health care (11 out of 33) and household (5 out of 33). In most studies, the sources of pneumonia case definition were physician’s diagnosis according to WHO IMCI definition (29 out of 34), culture–proved pneumonia was used for case definition in 1 study, self–reported condition according to WHO IMCI definition was adopted in one study, and two studies used health workers’ diagnosis. A number of different sources were used for gathering cost data, the most common being through hospital records and costing interviews/questionnaires. Interviews and questionnaires were commonly used in studies with a household perspective to collect data on indirect costs. Other sources included a national database of costs, insurance databases, surveillance data and pharmaceutical databases. The WHO CHOICE database, expert opinion and data from pilot studies were also used to collect data on the unit cost of pneumonia treatment.

The average quality score of 24 published studies was 8.21 out of 13 on scale based on modified Drummond checklist (range 3–12) (Appendix S5 in **Online Supplementary Document(Online Supplementary Document)**). The majority of the studies failed to consider discounting and did not perform sensitivity analyses. There were 6 studies considered high quality, 14 studies were medium quality, and 4 low quality studies. All studies were included into the final analysis.

### Cost of management per episode of pneumonia

**Figure 3:**
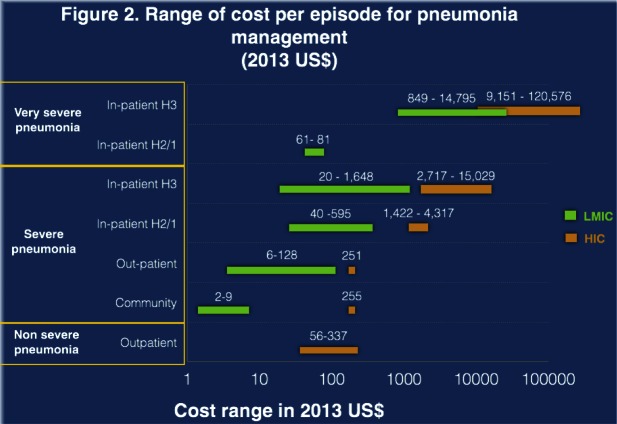
Range of cost per episode for pneumonia management (2013 US$).

Cost results stratified by income category are presented in **Table 2**, **Table 3** and **Figure 3**. In HICs, the mean cost of treatment for an episode of severe pneumonia at the hospital out–patient level was US$ 251.1 in Germany [16]. An Australian study [14] reported similar cost of US$ 254.9 for community management of severe pneumonia. Average costs of facility based case management for young children admitted in primary/sary and tertiary hospitals were US$ 2803.5 (95% CI IQR 2000.6–3683.3), and US$ 7037.2 (95% CI 4028.6–11 311.0) respectively, which was 11–28 fold higher than in those managed as out–patients. The total cost per episode for the management of non–severe pneumonia at out–patient level was also reported for three countries: US$55.8 in Uruguay [12], US$ 272.7 in Chile [12] and US$ 334.6 in the United States [36]. The cost for very severe pneumonia managed in general pediatric wards followed by intensive care unit (ICU) care was reported to be US$9151.3 in a tertiary hospital in Spain [17] and US$ 120 576.3 in the United States, which is nearly 2–17 fold of the cost for severe pneumonia management in hospital settings in HICs. The majority of studies from HIC took only direct medical costs into consideration. Only two studies included direct non–medical costs and indirect costs [16]. The mean proportion of the total cost for direct medical, direct non–medical and indirect costs were 41.5%, 19.5% and 38.5% respectively.

**Table 2:** Cost per episode for childhood pneumonia management in high–income countries

Severity	WHO region	Country, publication year	Perspective	Sample size	Cost per episode (2013 US$)				Cost component, % of total cost per episode		
**Tertiary/teaching hospital in–patient care**	**Secondary/primary hospital in–patient care**	**Out–patient care**	**Community care**	**Direct medical**	**Direct non–medical**	**Indirect**
**Non–severe pneumonia**	AMR	US, 2012*	Societal	940			334.6				
		Chile, 2007 [12]	Healthcare	366			272.7				
		Uruguay, 2007 [12]	Healthcare	366			55.8				
**Non–severe pneumonia mean cost (95% CI)**						**221.0 (55.8–334.6)**					
**Severe pneumonia by WHO IMCI Definition**	AMR	Chile, 2007 [12]	Healthcare	366		4316.7			100		
		Uruguay, 2007 [12]	Healthcare	366		1421.6			100		
		US, 2012*	Societal	940	15 029.2						
	EUR	North Ireland, 1999 [13]	NA	45	5733.8, 2716.8				100		
	WPR	Australia, 2011*	Societal	NA	6,259.1				93.1		6.9
**Hospitalised pneumonia**	WPR	Australia, 2008 [14]	Societal	528		2813.1		254.9	100		
		Australia, 2008 [15]	Healthcare	1348		2307.8			100		
	EUR	Germany, 2005 [16]	Societal	402		3158.6	251.1		41.5	19.5	38.5
		Spain, 2013 [17]	Healthcare	101	5447.3				100		
**Severe pneumonia mean cost (95% CI)**					**7037.2 (40 286–11 311.0)**	**2803.5 (2000.6–3683.3)**	**251.1**	**254.9**			
**Very severe pneumonia by IMCI**	AMR	US, 2012*	Societal	940	120 576.2						
**Very severe pneumonia require critical care**	AMR	Spain, 2013 [17]	Healthcare	101	9151.3						
**Very severe pneumonia mean cost (95% CI)**					**64 863.8 (9151.3–120 576.3)**						

NA – Information not available, EUR – Europe Region, AMR – The Americas Region, WPR – Western Pacific Region, CI – confidence interval, IMCI – Integrated Management of Childhood Illness

*Unpublished data.

**Table 3:** Cost per episode for childhood pneumonia management in low– and middle–income countries

Severity	WHO region	Country, publication year	Perspective	Sample size	Cost per episode (2013 US$)				Cost component, % of total cost per episode		
**Tertiary/teaching hospital in–patient care**	**Secondary/primary hospital in–patient care**	**Out–patient care**	**Community care**	**Direct medical**	**Direct non–medical**	**Indirect**
**Non–severe pneumonia**	SEAR	Viet Nam, 2010 [18]	Healthcare	788			28.6				
		Pakistan, 2008 [19]	Healthcare	141			29.4				
		Pakistan, 2006 [20]	Societal	502			94.1–17.8				
		Bangladesh, 2012*	Societal	340			5.7				
	AFR	Guinea, 1998 [21]	NA	73 650			3.2				
		South Africa, 2012 [22]	Societal/health care	745			263.1				
	AMR	Brazil, 2007 [12]	Healthcare	366			93.0				
**Non–severe pneumonia mean cost (95% CI)**						**66.9 (21.7–129.7)**					
**Severe pneumonia by WHO IMCI Definition**	SEAR	Pakistan, 2010*	Healthcare	NA				8.7	100		
		Pakistan, 2012 [23]	Household	423			7.9	1.5	89.1	1.3	9.6
		Bangladesh, 2012*	Societal	340			5.7				
		Bangladesh, 2010 [24]	Societal	360	193.6		124.0		Y	Y	
		Viet Nam, 2010 [18]	Healthcare	788		39.5			Y	Y	Y
		Pakistan, 2008 [19]	Healthcare	141		186.0			64.1	35.9	
		Pakistan, 2003 [25]	NA	126	20.3				100		
		Bangladesh, 2005*	Household	114	80.6†	62.6#			70.9†	29.1†	
		Bangladesh, 2010 [26]	Household	90	124.2				67.6	32.4	
		Indonesia, 2001*	Societal	NA	135.2				75	25	
	AFR	Guinea, 1998 [21]	NA	73650		110.6			69	30	
		South Africa, 2001*	Societal	509	480.9§	110.0					
	AMR	Brazil, 2007 [12]	Healthcare	366		461.0			100		
		Brazil, 2011*	Societal	79	1474.1†,‡	594.5#			94†	1†	5†
		Colombia, 2013 [27]*	Healthcare	1545		517.6			100		
		Argentina, 2012*	Societal	NA	1648.0				100		
**Hospitalised pneumonia**	SEAR	Viet Nam, 2001 [28]	Household	94				2.7	56–88	Y	
		Pakistan, 2006 [20]	Societal	502		310.8	127.6		45.3	55	
		India, 2009 [29]	Healthcare/household	56	145.7	44.7			45.7	5.3	47.4
		India, 2002 [30]	Societal	52	23.9				100		
	AFR	Zambia, 2009 [31]	Healthcare	9146		249.7	55.7		100		
		Kenya, 2009 [32]	Societal	205	236.8	162.1, 89.5			86	14	Y
		South Africa, 2011 [33]	Societal	509	491.4†, 1553.2‡				100		
		South Africa, 2012 [22]	Societal/health care	745	1223.1				98	2	0.2
	WPR	Fiji, 2012 [34]	Societal/household	390			25.7, 15.6		61.9	33.2	4.9
	AMR	Colombia, 2013 [27]	Healthcare	1545		304.4	76.2				
	EMR	Jordan, 2010 [35]	NA	728		563.4			100		
**Severe pneumonia mean cost (95% CI)**					**559.4 (268.9–886.3)**	**242.7 (153.6–341.4)**	**51.7 (17.4–91.0)**	**4.3 (1.5–8.7)**			
**Very severe pneumonia by IMCI**	SEAR	Bangladesh, 2012*	Societal	340			15.7				
		Viet Nam, 2010 [18]	Healthcare	788		61.2					
		Pakistan, 2008 [19]	Healthcare	141		81.3					
**Very severe pneumonia require critical care**	AFR	South Africa, 2011 [33]	NA	3014	849.0† 14795.4‡						
		South Africa, 2012 [22]	Societal/health care	745	6696.2						
	AMR	Colombia [27]	Healthcare	1545	3643.4						
**Very severe pneumonia mean cost (95% CI)**					**6496.0 (2246.2–12 007.4)**	**71.3 (61.2–81.3)**	**15.7 (15.7–15.7)**				

NA – information not available, Y – authors considered the cost component, but the proportion was unknown, EUR – Europe Region, AMR – The Americas Region, WPR – Western Pacific Region, SEAR – South East Asia, AFR – The Africa Region, EMR – Eastern Mediterranean Region

*Unpublished data.

†Public health care.

‡Private health care.

§Pediatric ward.

#Supplementary health system.

In LMICs, the cost of case management for severe pneumonia was reported across all treatment settings. The community management cost was only reported in studies conducted in South–East Asia region, with a mean cost of US$ 4.3 (95% CI 1.5–8.7) per episode. Out–patient care mean costs were US$ 51.7 (95% CI 17.4–91.0) per case. Costs for in–patient care varied by regions, level of hospitals (primary/sary/tertiary), and levels of care offered at a facility: the mean cost for primary/sary hospital care was 242.7 (95% CI 153.6–341.4) and for tertiary/teaching hospital was 559.4 (95% CI 268.9–886.3). Two groups–severe pneumonia by WHO IMCI definition and hospitalized pneumonia by physician’s diagnosis–showed similar costs in all levels of care. The in–patient care costs were 4–11 fold greater than that for out–patient care in the LMICs strata, which in turn was significantly higher than that for community management.

The costs for management of non–severe pneumonia managed at outpatient level were US$ 66.9 (95% CI 21.7–129.7), which was slightly higher than for severe cases at outpatient level. This is because the hospital short stay for non–severe pneumonia in South Africa [22] was relatively high at US$ 263.1 per episode. The mean cost for very severe cases was US$ 6496.0 (2246.2–12 007.4), which is nearly 10-fold of severe case management cost.

There were 6 studies reporting cost from household perspective, mainly from LMICs in South East Asia Region. Direct medical cost for severe pneumonia in hospital inpatient settings were 26.6%–115.8% of the monthly household income, thus demonstrating that severe pneumonia management in hospital placed a significant financial burden on families. On the other hand, outpatient and community management of severe pneumonia accounted for only 0.4%–4.1% of family’s monthly income indicating decreased burden in these settings. (**Table 4**).

**Table 4:** Direct medical cost for severe pneumonia management in low– and middle–income countries reported from household perspective

Country, publication year	Direct medical cost (2013 US$; % of direct medical cost to monthly household income)				Monthly household income (2013 $US)*	
	**Tertiary/teaching Hospital in–patient care**	**Secondary/primary hospital in–patient care**	**Out–patient care**	**Community ambulatory care**	
**Bangladesh, 2010 [**26**]**	124.2 (52.9%)	–	–	**–**	234.9
**Bangladesh, 2005**†	80.6^‡^ (34.3%)	62.6^§^ (26.6%)		**–**	234.9
**India, 2009 [**30**]**	305.8 (115.8%)	135.1 (64.7%)	–	**–**	264.0
**Pakistan, 2012 [**23**]**	–	–	7.9 (2.3%)	1.5 (0.4%)	338.3
**Viet Nam, 2001 [**28**]**	–	–	–	2.7 (0.7%)	398.6
**Fiji, 2012 [**34**]**	**–**	**–**	25.7 (4.1%)/15.6 (2.5%)	**–**	632.5‡

*Monthly household income (2013 US$) were derived from Gallup World Poll annual median household income, equals annual median household income divided by 12. These results were based on Gallup data gathered between 2006 and 2012 in 131 population. Source: http://www.gallup.com/poll/166211/worldwide-median-household-income-000.aspx.

†Numbers used GNI per capita based on purchasing power parity (PPP) due to lack of monthly household income data. Source: http://data.worldbank.org/indicator/NY.GNP.PCAP.PP.CD/countries.

‡Public health care.

§Supplementary health system.

Of the papers reporting cost component of severe pneumonia management, direct medical cost was reported across all studies and accounted for 45%–100% of the total cost. The mean direct non–medical cost and indirect cost were US$ 22.0 (11.8–32.7) and US$ 27.0 (4.0–54.3) respectively, which account for 0.5%–31.0% of weekly household income (**Table 5**).

**Table 5:** Direct non–medical cost and indirect cost per episode for severe pneumonia management in low– and middle–income countries

Country, publication year	Direct non–medical cost*			Indirect cost†	% of non–medical cost to monthly household income	Monthly household income (2013 US$)
	**Total**	**Transportation**	**Food**	**Total**
**Bangladesh, 2010 [**24**]**	32.4				13.8%	234.9
**India, 2002 [**29**]**	5.3	5.3		47.5	2.0%	264.0
**Pakistan, 2008 [**19**]**	35.9	12.2	23.7		10.6%	338.3
**Pakistan, 2006 [**20**]**	55.0				16.3%	338.3
**Pakistan, 2012 [**23**]**	3.3	2.3	1.0	9.6	1.0%	338.3
**Kenya, 2009 [**32**]**	14.0				9.0%	155.8
**Guinea, 1998 [**21**]**	30.0				31.0%	96.7‡
**South Africa, 2012 [**22**]**	2.0	1.4		0.2	0.5%	434.8
**Fiji, 2012 [**34**]**	33.2	33.2		4.9	5.3%	632.5‡
Brazil, 2011*	9.7	8.41	1.31	73.1	1.6%	626.8
**Mean (95% CI)**	**22.0 (11.8–32.7)**	**10.5 (3.5–22.3)**	**8.7 (1.0–23.7)**	**27.0 (4.0–54.3)**		
**Median (IQR)**	**22.0 (4.6 –33.9)**	**6.9 (2.1–11.5)**	**1.3 (–)**	**9.6 (2.5–60.3)**		

CI – confidence interval, IQR – interquartile range

*Direct medical costs include medications and consultation, non–medical cost includes transportation, food and accommodation.

†Indirect cost refers to parental loss of earnings in the period of illness. Monthly household income (2013 US$) was derived from Gallup World Poll annual median household income, equals annual median household income divided by 12. These results were based on Gallup data gathered between 2006 and 2012 in 131 populations. Source: http://www.gallup.com/poll/166211/worldwide-median-household-income-000.aspx.

‡Numbers used GNI per capita based on purchasing power parity (PPP) due to lack of monthly household income data. PPP GNI is gross national income (GNI) converted to international dollars using purchasing power parity rates. An international dollar has the same purchasing power over GNI as a US dollar has in the United States. GNI is the sum of value added by all resident producers plus any product taxes (less subsidies) not included in the valuation of output plus net receipts of primary income (compensation of employees and property income) from abroad. Source: http://data.worldbank.org/indicator/NY.GNP.PCAP.PP.CD/countries.

### Length of stay in hospital

The in–patient cost was determined primarily by the length of stay (LOS) and the average cost per bed day. In this review, we extracted length of stay for severe pneumonia for future costing analysis reference (**Tables 6–8**).

**Table 6:** Length of stay of very severe pneumonia and severe pneumonia in hospital in high–income countries

Country and year	Setting	LOS (SD) days	Sample size
**Very severe pneumonia:**			
Germany, 2005 [16]	ICU	7.4 (6.1)	2039
Spain, 2013 [17]	ICU	18.0	99
USA, 2012*	ICU	18.3 (43.1)	1116
Australia, 2011*	ICU	11.0	–
Median (IQR)		14.5 (10.1–18.1)	
**Severe pneumonia:**			
Ireland, 1999 [13]	Control group	8.3 (7.7–9.0)	44
	New treatment group	4.0 (3.5–4.6)	45
Germany, 2005 [16]	Hospitalised cases	7.4 (6.1)	2039
Australia, 2008 [14]	Without impact diary	8.8	202
	With impact diary	13.5	523
Australia, 2011*	Non–ICU	6.0	–
Spain, 2013 [17]	Non–ICU	10.5	99
USA, 2012*	Non–ICU	2.7 (2.3)	940
**Median (IQR)**		**7.9 (5.5–9.2)**	

IQR – interquartile range, ICU – intensive care unit

*Unpublished data.

**Table 7:** Length of stay of severe pneumonia in hospital in low– and middle–income countries

Country, year	Description	Length of stay (SD) in days‡	Sample size
**Viet Nam, 2010 **[18]	Probable pneumonia	7.2 (5.0)	40
	Radiograph confirmed	6.7 (3.8)	426
	Probable severe pneumonia	6.2 (3.3)	59
	Radiograph confirmed severe pneumonia	6.4 (2.7)	193
**Bangladesh, 2010 **[24]	Hospital care	6.0 (5.0–7.0)	180
Bangladesh, 2005*	Public health care	7.1	73
	Private health care	6.4	41
Bangladesh, 2010*	Hospital stay	7.0 (3.0)†	93
**Pakistan, 2003 **[25]	Antibiotic use duration	Approx. 8	124
**Kenya, 2009 **[32]	National hospitals	8.2	49
	District hospitals	6.7	30
	District hospitals	4.8	29
	District hospitals	4.2	17
	Provincial hospitals	6.6	31
	Mission Hospitals	7.8	30
	Mission Hospitals	3.4	19
**Zambia, 2009 **[31]	Tertiary health center	4.0	221
**Pakistan, 2008 **[19]	Time spent at health facility for severe pneumonia	3.3	65
**Pakistan, 2006 **[20]	Secondary hospital	3.0	502
**Jordan, 2010 **[35]	In–patient days	4.0–5.0	728
**India, 2009 **[30]	Secondary hospital	3.5 (2.9–4.1)	31
	Tertiary hospital	3.7 (3.0–4.4)	25
**India, 2002 **[29]	Tertiary hospital	6.5 (2.5)	52
Brazil, 2011*	Public health system	3.9 (2.2)	59
	Supplementary health system	5.3 (4.7)	20
**Colombia, 2013 **[27]	Primary	2.0 (1.0–2.0)	247
	Secondary hospital	4.0 (1.0–5.0)	1208
	Tertiary hospital	6.0 (3.0–9.0)	47
**South Africa, 2011 **[33]	Public sector ward	8.7	86
	Fee for service sector	5.6	7786
**South Africa, 2012 **[22]	Paediatric ward	8.1 (7.4–8.8)	513
Indonesia, 2001*	Non–ICU	6.7	–
Argentina, 2012*	Severe pneumonia	7.5(8.5)	42
	Unilateral focal pneumonia without complications	7.4 (6.0)	1994
	Multifocal pneumonia without complications	8.0 (6.5)	323
**Median (IQR)**		**6.4 (4.1–7.1)**	

ICU – intensive care unit

*Unpublished data..

†Combined HIV+ and HIV–, HIV+ had longer stay in ward (9.3 vs 7.0 days)

‡Length of stay (LOS) reported as mean, mean (standard deviation) or median (interquartile range). When stratified LOS available, then stratified LOS was reported, not average length of stay of all pneumonia.

**Table 8:** Length of stay of very severe and non–severe pneumonia in hospital in low– and middle–income countries

Country, year	Description	Length of stay (SD) days	Sample size
**Very severe pneumonia**			
Viet Nam, 2010 [18]	Very severe pneumonia	6.4(2.7)	26
	Confirmed very severe pneumonia	5.8 (3.0)	44
Colombia, 2013 [27]	ICU	13.0 (6.0–14.0)	43
South Africa, 2011 [33]	ICU	9.4	46
	ICU	10.5	93
South Africa, 2012 [22]	ICU	14.4(10.3–18.5)	7
Pakistan, 2008 [19]	Time spend at health facility for very severe pneumonia	3.9	35
Argentina, 2012†	Very severe pneumonia	8.9	–
	Unilateral focal pneumonia without complications	17.2	–
	Multifocal pneumonia without complications	11.5	–
Brazil, 2011*	Public health system	6.9	–
	Supplementary health system	6	–
Median (IQR)		9.2 (6.1–12.6)	
**Non severe pneumonia**			
Pakistan, 2008 [19]	Time spend at health facility for pneumonia	0.3	41
South Africa, 2012 [22]	Short stay	1.4 (1.3–1.6)	338
**Median (IQR)**		**0.9 (0.3–1.4)**	

ICU – intensive care unit, IQR – interquartile range

*Unpublished data.

†Note added in proof: The data from this study are unpublished but the data on the length of stay are published in Giglio ND, Cane AD, Micone P, Gentile A. Cost-effectiveness of the CRM-based 7-valent pneumococcal conjugated vaccine (PCV7) in Argentina. Vaccine. 2010;28:2302-10. Medline:20064478.

The mean LOS for severe pneumonia reported in individual studies ranged from 4–13.5 days, with a mean LOS 7.7 (95% CI 5.5–9.9) days and median 7.9 (IQR 5.5–9.2) days in HIC, and mean LOS 5.8 (95% CI IQR 5.3–6.4) days and median 6.4 (IQR 4.1–7.1) days in LMIC. For very severe pneumonia management in intensive care unit (ICU), LOS ranged from 7.4 to 18.3 days. The mean and median LOS were 13.7 (95% CI IQR 9.2–18.2) and 14.5 (IQR 10.1–18.1) days in HIC, and 9.5 (95%CI, 7.4–11.8) and 9.2 (IQR 6.1–12.6) days in LMIC.

### Unit cost of case management

Unit cost of treatment and resource uptake should be routinely reported in cost studies. However, only 13 of the 34 included studies reported these data. Since treatment protocols (use of antibiotics, diagnostic tests, procedures and levels and intensity of care) varied between studies, this contributed to variations in costs across studies. For example, the average cost of chest radiograph in LMIC was US$ 8.4 (95% CI 4.3–27.0), which was significantly lower than US$ 185.5 (95% CI 66.3–357.7) in high income countries (**Table 9**). We attempted to abstract unit cost data but were unable to include it in the presented direct medical costs because of paucity of information.

**Table 9:** Chest Radiography cost per episode

Country, year		Cost per episode (2013 US$)
**High income countries**	Australia, 2011*	129.8
	Chile, 2007 [12]	135.1
	Uruguay, 2007 [12]	43.4
	United States, 2012*	433.7
**Mean(SD)**		**185.5 (66.3–357.7)**
**Median(IQR)**		**132.5 (108.2–209.8)**
**Low– and middle–income countries**	Argentina, 2012*	26.7
	Brazil, 2011*	10.7
		6.0
	Brazil, 2007 [12]	13.63
	Bangladesh, 2010*	2.3
	India, 2009 [30]	5.4
	Pakistan, 2008 [19]	3.2
	Indonesia, 2001*	4.6
	Kenya, 2009 [32]	2.3
	South Africa, 2001*	29.7
	South Africa, 2011 [33]	59.7
		137.2
	South Africa, 2012 [22]	27.7
**Mean (SD)**		**25.3 (9.8–47.3)**
**Median (IQR)**		**8.4 (4.3–27.0)**

*Unpublished data.

## DISCUSSION

This is the first attempt to conduct a systematic review of all published and available unpublished cost data on the management of childhood pneumonia. Costs per episodes in HICs were 5–13–fold higher in all delivery channels than those in LMICs. The review demonstrates that the magnitude of cost per episode increases markedly as the level of treatment delivery rises. Community management for severe pneumonia was less than 10% that of the cost of out–patient management among all levels of management in LMICs. Thus, there are strong economic reasons for considering community case management as a central strategy for pneumonia case management in low income countries; this merits further evaluation which should include consideration of medical outcomes. The mean lengths of stay in hospital for severe pneumonia were 1.8–4.6 days less in LMIC compared to HIC, and at a mean of 5.8 and median of 6.4 days, were close to the WHO recommendation of 5 days in–patient treatment [10].

We demonstrated that the cost (per episode) for the management of severe pneumonia varied greatly by unit cost of intervention, disease severity and treatment procedures in different settings. The review also demonstrated that major factors governing the total cost per episode were length of stay in the hospital, countries income level and the presence or absence of community case management for pneumonia. Many other studies have also found GDP per capita to be the main driver of costs [37]. These findings demonstrate that choosing the appropriate value for these inputs will have a significant influence on the total cost. Existing studies calculated pneumonia management costs in many countries assuming the same treatment procedure and unit cost of medicine. However, the cost data we collected demonstrate that this method may have limitations; the uncertainty in the traditional estimates can be measured using the cost data reported in this review.

Our results showed that direct medical costs for childhood pneumonia management, especially inpatients, represent a significant proportion of the average monthly household income for families in LMICs. This is often compounded by further direct non–medical cost and indirect cost ie, loss of earnings when caring for the sick child. In countries where these families were uninsured, health payments for pneumonia management were a heavy burden on household and can have a significant impact on the family, particularly when the payments for care were out–of–pocket in most LMIC countries. Alamgir et al investigated the impact that this strain had on families and how they source the funds: many borrow or take high–interest loans [26]. Furthermore, Ayieko et al found that 10% of the patients in district hospitals and up to 25% of children in tertiary hospitals wait in hospital beds after medical discharge while families source the fees. The latter translates to an additional cost of US$ 17.46 to the public provider and US$ 5.32 to the family [32], resulting in a drain on both the resources of the family and the health care provider, as well as denying a bed to another sick child. It is therefore important that national strategies for pneumonia management in LMIC are not only cost–effective for the national program but also give attention to the burden of costs on families so that these are maintained at a level that is affordable.

The data in this review comprise “actual” cost data measured in cost studies conducted in many LMIC and HIC. We believe that these represent a fair first approximation of true costs in these countries. It is noteworthy that the resulting cost estimates are higher than those currently contained in the WHO–CHOICE estimates [38]. Three factors could have contributed to this variation. First, we identified longer facility and hospital stays compared to standard treatment protocols recommended by the WHO [39]. Moreover, most existing cost studies were conducted at tertiary level hospitals where out–patient and in–patient treatments carry a much higher cost compared to the community or first level facility. Third, the wide variety of antibiotics (including variations in dosage, route of administration and duration) across the sites, as well as the heterogeneity in the costing methodology and the cost components in existing studies may have led to higher estimates.

This review has several limitations. First, the primary goal of the systematic review was to obtain data on cost of management (per episode) of severe pneumonia. However, the lack of any standard management protocols (which varied widely across the included studies) and the general lack of service uptake data, may have contributed to the substantial uncertainty around the estimates. Second, we did not include costs of diagnostic investigations in the cost modeling in some study sites, because country–specific unit prices and utilization data were not available. Therefore, the true economic burden resulting from the management of childhood pneumonia could be considerably higher. Third, costs were highly dependent on level of care offered at facility and LOS could be skewed to longer period if high level of care (such as intensive care unit (ICU) care) was offered to severe and very severe cases. In this review, severe cases were all managed at non–ICU hospital settings, and very severe cases were managed at both non–ICU and ICU care in hospital settings. We were able to report LOS separately for non–ICU and ICU care but this stratification was not possible for total cost per episode. A further limitation was that the definition of ICU and ICU care may vary by country. Fourthly, we limited the search to English articles only, which may exclude some cost reported in other languages, however only 13 studies out of 789 articles in other languages were found. We tried to compliment this with unpublished data from non–English speaking countries. Lastly, there was a wide range in per capita income and health care system and payment schemes within LMIC category and the existing cost data may only reflect the situation when and where the data were collected and may not be representative of the whole country or the current situation. Furthermore, there were not sufficient studies to address all possible cost scenarios to facilitate international and public/private comparisons.

Our results demonstrate that further research on the economic burden due to the management of childhood pneumonia is needed, with clear reporting of data on unit cost of intervention, dosage of various drugs and information on health care utilization, such as length of stay in hospital. We recommend that standard reporting of unit cost of intervention with direct medical and non–medical costs and indirect costs, standard treatment protocols and health resource utilization in conjunction with the total cost per episode in any cost–of–illness studies would facilitate economic estimates of national scale–up and international comparisons. Further studies on the cost–effectiveness of standardized IMCI protocol against other treatment protocols could be expected to find a cost–saving management strategy for high burden countries.

Identifying the most cost–effective interventions for pneumonia management is essential for achieving the goal of further reducing child mortality. Our study demonstrated that early treatment in the community costs less (per event) than late treatment in the hospital. This finding suggests that the public health community should explore ways for community outreach for early diagnosis and treatment before severe pneumonia sets in. The results from this systematic review provide important missing information on the cost of pneumonia treatment in children across many countries. These data and the cost estimates should provide important information useful to program managers and policy makers at national and regional levels, international agencies, and donor organisations to aid resource allocation, program planning and priority setting. The estimates presented in this review could enable a more detailed economic evaluation of the revised WHO pneumonia management guidelines [39], and help identify the most cost–effective preventive and treatment interventions for reducing the burden of childhood pneumonia.

## 

**Table 1:** Characteristics of all studies included*

WHO region	Country, publication year	Study population	Healthcare setting	Severity of pneumonia studied	Study design	Source of case definition	Perspective	Sample size	Mean (SD) /median age of patients (months)	Data source
**High–income countries (number of studies = 8)**										
EUR	Northern Ireland, 1999 [39]^1^	Antrim (urban)	H2	S	QES	PD	N/A–	45	39.60 (16.8)	H
	Spain, 2013 [17]	Barcelona (urban)	H3	S, VS	Cost analysis‡	Culture–proved pneumonia	Healthcare	101	39.60	H
	Germany, 2005 [16]	National	O,H1	S, VS	Cost–of–illness	PD	Societal	402	N/A	N, IQ
AMR	Chile, Uruguay, 2007 [12]	National	O,H1	S, NS	Cost analysis‡	PD, ICD–10	Healthcare	366	N/A	H,IQ
	United States, 2012*	Denver, Colorado (urban)	H3	S, VS, NS	Cost–of–illness	PD by WHO IMCI definition	Societal	940	0–59	H, P
WPR	Australia, 2008 [15]	National	O, H1–3	S	Cost analysis‡	ICD–10	Healthcare	1348	N/A	N
	Australia, 2008 [14]	Melbourne, Victoria (urban)	O,H1	S	Cohort study/cost–of–illness	Health professional’s diagnosis	Societal	528	N/A	N,H,IQ, Pilot
	Australia, 2011*	Sydney (urban)	H3	S, VS	Cost–of–illness	PD by WHO IMCI definition	Societal	N/A	N/A	P, Market price
**Low– and middle–income countries (number of studies = 27)**										
SEAR	Bangladesh, 2010 [26]	Dhaka (urban)	H3	S	Cost–of–illness	PD	Family	90	5.00	IQ
	Bangladesh, 2005†	Dhaka (urban)	H3	S, VS	Cost–of–illness	PD by WHO IMCI definition	Household	114	70.32	IQ
	Bangladesh, 2010 [24]	Mirpur, Dhaka (urban)	O, H2	S	RCT/CEA	PD by WHO IMCI definition	Societal	360	8.00	–
	Bangladesh, 2010†	Barishal, Bogra, Comilla, Kishoregonj (urban)	H3	S, NS	Cost–of–illness	PD by WHO IMCI definition	Societal	235	N/A	IQ
	Bangladesh, 2012†	Mohakhali, Dhaka (urban)	H3	S, VS, NS	Cost–of–illness	PD by WHO IMCI definition	Societal	340	N/A	H
	India, 2009 [30]	Vellore (rural)	H1, H2	S	Cost–of–illness	PD by WHO IMCI definition	Healthcare/ Household	56	8.8	H, IQ
	India, 2002 [29]	Berhampur, Orissa (urban and rural)	H3	S	Epidemiological study	PD	Societal	52	N/A	H, IQ
	Indonesia, 2001†	Lombok (rural)	H3	S	Cost–of–illness	PD by WHO IMCI definition	Societal	N/A	N/A	H
	Pakistan, 2003 [25]	Peshawar city (urban)	H3	S	RCT/CEA	PD by WHO IMCI definition	–	126	N/A	–
	Pakistan, 2006 [20]	Ghizer district (rural)	O, H1, H2	S, NS	Cost analysis‡	PD	Societal	502	N/A	IQ
	Pakistan, 2008 [19]	Ghizer district (rural)	O, H1, H2	S, VS, NS	Cost analysis‡	PD by WHO IMCI definition	Healthcare	141	N/A	IQ
	Pakistan, 2010†	Matiari (rural)	C	S	Cost–of–illness	PD by WHO IMCI definition	Healthcare	N/A	N/A	Surveillance
	Pakistan, 2012 [23]	Haripur district (rural)	C, H1, H2	S	Cost analysis‡	WHO definition by health worker	Household	423	N/A	H, IQ
	Viet Nam, 2010 [18]	Nha Trang city (urban)	H2	S, VS, NS	Cost–of–illness	PD by WHO IMCI definition	Healthcare	788	12.67	N, H
	Viet Nam, 2001 [28]	Ba Vi district (rural)	C, O, H1	S	Cost analysis‡	WHO definition, self–reported	Household	94	N/A	IQ
AFR	Guinea, 1998 [21]	National	O, H1	S, NS	CEA	PD	–	73650	N/A	H, E
	South Africa, 2011 [33]	Pretoria (urban)	H3	S, VS	Cost analysis‡	WHO definition	–	3014	N/A	H
	South Africa, 2012 [22]	National	H3	S, VS, NS	RCT	PD	Societal/health care	745	N/A	H, IQ
	South Africa, 2001†	Soweto (urban)	H3	S, VS	Cost–of–illness	PD by WHO IMCI definition	Societal	509	14.00	H,IQ
	Kenya, 2009 [32]	National	H3, H2, H1	S	Cost analysis‡	PD	Societal	205	12.00	H, IQ
	Zambia, 2009 [31]	Kanyama Township (urban)	O,H2	S	Cost analysis‡	PD	Healthcare	9146	N/A	N,H,P,W
AMR	Colombia, 2013 [27]	National	H1,H2,H3	S, VS, NS	Cost–of–illness§	WHO definition, radiographically diagnosed	Healthcare	1545	N/A	I
	Brazil, 2011†	Goiânia (urban)	H3	S, VS	Cost–of–illness§	PD by WHO IMCI definition	Societal	79	0–36	H, N
	Argentina, 2012†	Buenos Aires (urban)	H3	S, VS	Cost–of–illness§	PD by WHO IMCI definition	Societal	N/A	N/A	N
	Brazil,2007 [12]	National	O,H1	S, NS	Cost analysis‡	PD, ICD–10	Healthcare	366	N/A	H,IQ
WPR	Fiji, 2012 [34]	Viti Levu (urban and rural)	O	S	Cost analysis‡	PD by WHO IMCI definition	Societal/household	390	N/A	N,H, IQ
EMR	Jordan, 2010 [35]	Amman	H1	S	Cohort study	PD	–	728	4.30	N/A

*Severity of pneumonia: NS – non severe, S – severe, VS – very severe. Data source: H – hospital records, N – national data, IQ – interviews and questionnaires, I – insurance database, P – pharmacy database, W – WHO database. Treatment settings: H3 – tertiary hospital in–patient, H2–secondary hospital in–patient, H1 – primary hospital inpatient, O – out–patient care, C – community ambulatory care; PD – physician’s diagnosis, CEA – cost effectiveness analysis, RCT – randomized clinical trial, QES– quasi–experimental study, N/A – not available, PD – physician’s diagnosis, IMCI – Integrated Management of Childhood Illness, WHO – World Health Organization, SD – standard deviation.

EUR – Europe Region, AMR – the Americas Region, WPR – Western Pacific Region, SEAR – South East Asia, AFR– The Africa Region, EMR– Eastern Mediterranean Region

†Unpublished data.

‡The analysis of the comparative costs of alternative treatments or health care programmes.

§The cost analysis of treatment of a disease.
