# Supplementary material for: Cost of management of severe pneumonia in young children: systematic analysis
Source: J Glob Health. 2016 Mar 15;6(1):010408. doi: 10.7189/jogh.06.010408 (PMC4871066; doi:10.7189/jogh.06.010408)
Supplement: Online Supplementary Document [file jogh-06-010408-s001.pdf]

Online Supplementary Document

Zhang et al. Cost of management of severe pneumonia in young children: systematic analysis

J Glob Health 2016;6:010408

Appendix 1 Search Strategy

1) MEDLINE (via Ovid®): *1948 to October 2013*

No.	Searches
1	exp Pneumonia/
2	lrti.tw.
3	alri.tw.
4	ari.tw.
5	pneumonia$.tw.
6	1 or 2 or 3 or 4 or 5
7	exp Therapeutics/
8	treat$.tw.
9	(care adj3 in–patient$).tw.
10	(care adj3 out–patient$).tw.
11	(care adj3 hospital$).tw.
12	(care adj3 communit$).tw.
13	7 or 8 or 9 or 10 or 11 or 12
14	exp Economics/
15	cost$.tw.
16	econom$.tw.
17	expend$.tw.
18	14 or 15 or 16 or 17
19	6 and 13 and 18
20	limit 19 to ("all infant (birth to 23 months)" or "preschool child (2 to 5

2) EMBASE (via Ovid®): *1980 to October 2013*

No.	Searches
1	exp pneumonia/
2	lrti.tw.
3	alri.tw.
4	ari.tw.
5	pneumonia$.tw.
6	1 or 2 or 3 or 4 or 5
7	exp therapy/
8	(care adj3 in–patient$).tw.
9	(care adj3 out–patient$).tw.
10	(care adj3 hospital$).tw.
11	(care adj3 communit$).tw.
12	treat$.tw.
13	7 or 8 or 9 or 10 or 11 or 12
14	exp economic aspect/
15	cost$.tw.
16	econom$.tw.
17	expend$.tw.
18	14 or 15 or 16 or 17
19	6 and 13 and 18
20	limit 19 to (infant <to one year> or preschool child <1 to 6 years>)

3) DARE, NHS EED & NHS HTA (via CRD – www.crd.york.ac.uk): *All records to October 2013*

No.	Searches
1	MeSH Pneumonia EXPLODE 1 2
2	pneumonia*
3	lrti
4	alri
5	ari
6	#1 OR #2 OR #3 OR #4 OR #5
7	MeSH Therapeutics EXPLODE 1
8	treat*
9	therap*
10	#7 OR #8 OR #9
11	MeSH Economics EXPLODE 1 2
12	cost*
13	econom*
14	expend*
15	#11 OR #12 OR #13 OR #14
16	MeSH Child, Preschool EXPLODE 1
17	MeSH Infant EXPLODE 1
18	#16 OR #17
19	#6 AND #10 AND #15 AND #18

4) The Cochrane Library (via Wiley Online Library – onlinelibrary.wiley.com). *All records to October 2013*

No.	Searches
1	MeSH descriptor Pneumonia explode all trees
2	pneumonia*
3	lrti
4	alri
5	(#1 OR #2 OR #3 OR #4)
6	MeSH descriptor Therapeutics explode all trees
7	treat*
8	therap*
9	(#6 OR #7 OR #8)
10	MeSH descriptor Economics explode all trees
11	cost*
12	econom*
13	expend*
14	(#10 OR #11 OR #12 OR #13)
15	MeSH descriptor Child, Preschool explode all trees
16	MeSH descriptor Infant explode all trees
17	(#15 OR #16)
18	(#5 AND #9 AND #14 AND #17)

Appendix 2. Inclusion and exclusion criteria for published studies.

Inclusion required ALL of the following:	Exclusion if ONE of the following was true:
• English language<br>• Empirical cost data presented<br>• All–cause pneumonia<br>• Treatment of pneumonia using antibiotics<br>• Reporting specifically for children aged 0–59 months<br>• Unbiased population sample	• “Vaccine–“ or “Palivizumab” trials<br>• Specially selected cohorts<br>• “Non–standard” interventions<br>• Review articles and conference abstracts

Appendix 3 Modified Drummond checklist for quality assessment for published studies

1	The question and perspective are clearly stated
2	It is a cost–study
3	Quantity of resources used and unit costs are reported separately
4	Data sources are clearly reported
5	Currency & Price (including price year) are documented
6	Time horizon of costs is stated
7	Consideration of discounting is documented and justified
8	Details of statistical tests and result errors are clearly described
9	Sensitivity analysis is carried out (and adjustments stated)
10	A comparison is made between two alternatives
11	A standardised definition of pneumonia is used (ICD/IMCI/WHO etc)
12	The answer to the study question is clearly stated (and valid)
13	Conclusions are drawn, and relevant limitations are raised.

Appendix 4 Costing spreadsheet and practical guide (see separate file attached)

Appendix 5 Results of quality assessment score

Authors	Year	Quality Score	Unaddressed checklist items
**Al–Eidan,et al (22)**	1999	8	2,3,7,9,11
**Alamgir, et al (18)**	2010	7	3,5,7,9,10,11
**Anh,D et al (31)**	2010	12	10
**Ashraf,et al (24)**	2010	8	2,3,4,7,9,
**Aurangzeb & Hameed (27)**	2003	9	7,8,9
**Ayieko et al (19)**	2009	11	7,11
**Chola et al (35)**	2009	8	7,8,9,11,12
**Constenla,D. (14)**	2007	10	7,8,9
**Ehlken et al (12)**	2005	9	5,7,9,11
**Hussain,et al (29)**	2008	8	1,3,7,8,9
**Hussain,et al (28)**	2006	7	3,6,7,8,9,11
**Jha et al (33)**	1998	8	2,3,6,9,11
**Khuri–Bulos,et al (38)**	2010	3	1,2,3,4,5,7,8,9,11,12
**Lambert et al (13)**	2008	6	1,2,3,7,9,11,12
**Madsen,et al (25)**	2009	11	7,9
**Newall & Scuffham (23)**	2008	9	3,7,8,10
**Sahu,et al (26)**	2002	3	2,3,4,5,7,8,9,10,11,13
**Toan,et al (32)**	2001	6	3,5,7,8,9,11,13
**Temple et al (37)**	2012	11	7,9
**Sadruddin et al (30)**	2012	8	3,5,7,8,9
**Alvis–Guzman et al (36)**	2013	8	3,6,7,9,10
**Brotons et al (15)**	2013	8	3,6,7,9,11
**Kichin et al (34)**	2011	8	3,5,6,7,9
**Sinha et al (16)**	2012	11	9,11
	Mean	8.21	
